# Bridging clinical and environmental reservoirs: antimicrobial resistance in the emerging pathogen *Shewanella algae*

**DOI:** 10.1128/aac.01891-25

**Published:** 2026-04-15

**Authors:** Celia García-Rivera, Juan J. Roda-Garcia, Juan Carlos Rodríguez, Carmen Molina-Pardines, Iryna Tyshkovska, Jose M. Haro-Moreno, Antonio Martínez-Murcia, Maria Paz Ventero, Mario López-Pérez

**Affiliations:** 1 Microbiology Department, Alicante University General Hospital, Alicante Institute of Sanitary and Biomedical Research (ISABIAL), Alicante, Spain; 2 Microbial Genomics and Evolution Group, División de Microbiología, Universidad Miguel Hernández, Alicante, Spain; 3 Genetic PCR Solutions, Orihuela, Spain; Universita degli Studi di Roma "La Sapienza", Rome, Italy

**Keywords:** *Shewanella algae*, antimicrobial resistance, antibiotic resistance, pathogen emergence, mobile genetic elements, One Health, mobile integron

## Abstract

The emergence of antimicrobial resistance in environmental bacteria threatens therapeutic efficacy in clinical settings. *Shewanella algae*, historically regarded as a marine saprophyte, is increasingly recognized as an emerging opportunistic pathogen. In this study, we analyzed 86 *S. algae* isolates from Spain (19 clinical and 67 environmental) and integrated them with 178 publicly available genomes to explore antimicrobial susceptibility patterns and genomic diversity. Penicillins and fosfomycin consistently showed poor activity, whereas piperacillin/tazobactam, third- and fourth-generation cephalosporins, aminoglycosides, ciprofloxacin, trimethoprim–sulfamethoxazole and several novel β-lactam–inhibitor combinations exhibited low MIC distributions. Recently introduced agents, including ceftazidime/avibactam, ceftolozane/tazobactam, and cefiderocol, also demonstrated strong *in vitro* activity. Carbapenems displayed an unusual intraclass pattern, with imipenem showing markedly higher MICs than meropenem and ertapenem. When interpreted using CLSI’s “Other Non-Enterobacterales” criteria, clinical and environmental isolates exhibited largely overlapping susceptibility profiles, highlighting the potential role of environmental strains as reservoirs of resistance-related traits. Genomic profiling revealed a conserved intrinsic resistome (OXA-type β-lactamases, *qnrA* variants, *ugd*, and efflux regulators) together with horizontally acquired determinants. A 29 kb genomic island carrying multiple resistance genes was identified in a clinical isolate, with homologous structures detected in *Vibrio* and *Proteus*, suggesting interspecies transfer. Furthermore, plasmids harboring class 1 integrons (mobile integrons) were widespread, shared with Enterobacterales and *Vibrionaceae* across clinical and environmental settings. Overall, these findings highlight *S. algae* as both a clinically relevant pathogen and a reservoir of mobile AMR determinants and underscore the urgent need for species-specific antimicrobial susceptibility interpretive criteria to improve clinical decision-making for this emerging pathogen.

## INTRODUCTION

The emergence of novel and reemerging pathogens is a growing global public health concern (1). Environmental microorganisms, traditionally considered non-pathogenic, are increasingly associated with human infections, driven by ecological and social factors such as rising sea surface temperatures, coastal urbanization, intensification of aquaculture, international travel, and the globalized trade of seafood (2). These conditions not only expand the geographical range of many marine and freshwater bacteria but also increase opportunities for human exposure through recreational activities, consumption of raw or undercooked seafood, and contact with contaminated water. Emerging pathogens are typically defined as microorganisms whose incidence in humans has increased within the past two decades or threatens to increase in the near future. Among aquatic gram-negative bacilli, *Vibrio vulnificus* has gained prominence, particularly in the Gulf of Mexico, the coast of Florida (USA), and northern European waters, where warming seas have been linked to its rising incidence in severe wound infections and septicemia (3, 4). Similarly, *Aeromonas hydrophila* has been implicated in gastrointestinal and soft tissue infections, with higher case numbers reported in tropical and subtropical regions as well as in immunocompromised hosts (5). Other examples include *Vibrio parahaemolyticus*, a major cause of seafood-borne gastroenteritis worldwide (6), and *Mycobacterium marinum*, which causes skin infections (“fish tank granuloma”) in individuals exposed to aquaria or marine environments (7). Within this expanding group of opportunistic aquatic pathogens, *Shewanella algae* has recently emerged as a clinically relevant species. First described in the 1980s, it has since been increasingly reported across Asia, Europe, and the Americas, underscoring its pathogenic potential and raising concern regarding its antimicrobial resistance (8).

*S. algae* is a facultatively anaerobic, gram-negative, motile bacillus commonly found in brackish and marine environments. Although initially regarded as a saprophytic organism, its clinical relevance has become increasingly apparent over the past three decades (9, 10). Its remarkable metabolic versatility enables survival across diverse ecological niches, including extreme conditions, through the ability to use a wide range of electron acceptors such as metals and sulfur compounds (11). In humans, *S. algae* has been associated with a range of infections, including skin and soft tissue infections, otitis, bacteremia, and, less frequently, invasive systemic disease (12, 13). These infections most often occur in individuals with underlying conditions or after exposure to contaminated water or seafood, with a higher incidence in warm and temperate climates (14). A characteristic clinical manifestation is the so-called “patera foot syndrome,” described among migrants arriving by sea in overcrowded vessels, where prolonged exposure to seawater and poor hygienic conditions lead to necrotizing soft tissue infections (15, 16). Such cases highlight the pathogenic potential of *S. algae* under conditions of host vulnerability and environmental stress.

Despite its growing clinical importance, the pathogenic mechanisms of *S. algae* remain poorly understood, and its accurate identification is hampered by frequent misclassification with *Shewanella putrefaciens* when only conventional methods are applied. MALDI-TOF mass spectrometry has improved species-level recognition, although 16S rRNA sequencing and/or whole-genome analysis is still required in doubtful cases (17). A major concern is the lack of species-specific clinical breakpoints for these microbes in most international guidelines, which complicates the interpretation of antimicrobial susceptibility testing (AST) and limits standardized therapeutic decision-making. This gap is particularly worrisome given the increasing reports of multidrug-resistant *S. algae* strains, raising the prospect of treatment failures and underscoring its potential impact as an emerging public health threat (18).

To date, systematic studies on the *in vitro* activity of antimicrobials against *S. algae* are limited, and most derive from Asia, where epidemiological trends and resistance profiles may differ substantially from those in Europe (19–21). Reports from aquaculture and clinical settings describe multidrug-resistant strains carrying determinants against β-lactams, trimethoprim, tetracyclines, quinolones, and colistin, as well as efflux pump systems (22). These findings support the notion that *Shewanella* species may act as reservoirs of transferable resistance genes (18). More recent epidemiological studies indicate an increasing number of *Shewanella* infections with concerning resistance profiles, particularly involving carbapenems, in southern Europe (9, 13). These observations are consistent with the organism’s ecological preference for warmer environments and highlight the role of environmental and demographic factors in its emergence. However, there is still a critical lack of updated local data from southern Europe to guide empirical therapy. In this context, studies evaluating the susceptibility profile of *S. algae* using currently available interpretive frameworks are essential to support clinical decision-making in the absence of species-specific breakpoints. Such data are urgently needed to optimize therapeutic management, prevent treatment failures, and inform the development of evidence-based recommendations for this emerging pathogen.

Therefore, this study pursued two main objectives: first, to expand the database of *S. algae* isolates by incorporating a large number of clinical and environmental strains from the same region, thereby enabling robust comparative analyses; second, to characterize the antimicrobial resistance profile of *S. algae* in southern Europe by integrating phenotypic antimicrobial susceptibility testing*,* interpreted according to the Clinical and Laboratory Standards Institute (CLSI) “Other Non-Enterobacterales” criteria, with genomic screening of resistance determinants, and to provide exploratory epidemiological data that may support the future definition of species-specific interpretive thresholds. By bridging phenotypic and genotypic data, this work seeks to advance the understanding of antimicrobial resistance in *S. algae* and to support the development of more reliable diagnostic tools and evidence-based therapeutic strategies for infections caused by this emerging pathogen.

## MATERIALS AND METHODS

### Study design and isolate collection

A total of 86 *S. algae* isolates, obtained from both clinical and environmental sources, were included in this study (Table S1). Clinical isolates were collected from patients admitted to different hospitals in the Valencian Community and submitted to the Microbiology Service of the General University Hospital Dr. Balmis (Alicante, Spain) through the REDMIVA (*Red de Vigilancia Microbiológica de la Comunidad Valenciana*) and the Hospital Universitario Marqués de Valdecilla (Santander, Spain). Isolates were stored at −80°C in tryptic soy broth supplemented with 15% glycerol until analysis. Environmental isolates were obtained from sediment and seawater samples collected near the coast of Alicante (Spain; 38° 26.665′ N; 0° 22.364′ W). Sampling was carried out weekly during the summer of 2024, when the average surface water temperature was approximately 26°C. Samples were collected aseptically: surface seawater was collected in sterile high-density polyethylene bottles, while surface sediments were sampled using sterile 50 mL tubes. All samples were transported to the laboratory at ambient temperature and processed immediately upon arrival. For sediment samples, approximately 5 g were resuspended in sterile phosphate-buffered saline (PBS; 0.01 M, pH 7.4), vigorously shaken, and allowed to settle. A 1 mL aliquot of the supernatant was used for downstream processing. For seawater samples, a 1 mL aliquot was directly collected and diluted in PBS. Aliquots (100 μL) of each dilution and of the undiluted samples were plated onto Lyngby iron agar supplemented with 0.04% (wt/vol) L-cysteine. Although this medium is not fully selective for *Shewanella* spp., it allows recovery of H_2_S-producing colonies, which appear black because of iron sulfide deposition derived from the reduction of thiosulfate or L-cysteine, a phenotypic trait shared by all *Shewanella* species (23). Inoculated plates were incubated at 32°C for 24 h. Colonies displaying black precipitates were selected and purified through three consecutive subcultures on the same medium. Purified isolates were subsequently preserved and processed with the same downstream workflows used for clinical isolates.

### Species identification and whole-genome sequencing

Initial identification was performed using MALDI-TOF mass spectrometry (Bruker Daltonics) according to the manufacturer’s criteria. Confirmation was obtained using (i) 16S rRNA gene sequencing of the V3–V4 hypervariable region (24, 25) and (ii) whole-genome sequencing using long-read GridION technology (Oxford Nanopore Technologies, Oxford, UK). All clinical and environmental isolates identified as *S. algae* were subjected to whole-genome sequencing without any prior selection. Genomic DNA was extracted using GeneJET Genomic DNA Purification Kit (Thermo Fisher Scientific, Waltham, MA, USA). The quantity and integrity of genomic DNA were assessed using a Qubit fluorometer (Thermo Fisher Scientific) and electrophoresis on a 0.8% (wt/vol) agarose gel. High-molecular-weight DNA was sheared with g-TUBE (Covaris, Woburn, MA, USA) according to the manufacturer’s instructions to obtain fragments with an average size of 10–15 kb. Sequencing libraries were prepared with the Ligation Sequencing Kit (SQK-LSK109) in combination with the Native Barcoding Kit (SQK-NBD104). Up to 18 barcoded genomes were multiplexed per R10.4 flow cell and sequenced on a GridION system (Oxford Nanopore Technologies). Raw signal data were basecalled using Guppy v6.3.2 (Oxford Nanopore Technologies). All genomes achieved high sequencing depth (>50× and up to ~100× ) despite multiplexing, ensuring robust consensus accuracy for R10.4 long-read data. *De novo* assemblies were generated using Flye v2.9 (26), polished with Medaka(27, 28) and evaluated for completeness and contamination using CheckM2 (29). Taxonomic assignment was performed using the Genome Taxonomy Database (GTDB) v2.4.0 (28).

### Comparative genomics

To contextualize local isolates, all publicly available *S. algae* genomes were retrieved from NCBI as of February 2025, based on GTDB classification (30) (Fig. S1). Genomes with fewer than 100 contigs, completeness >90%, and contamination <5%, as assessed by CheckM2 (31), were included for analysis (Tables S1 and S2). Pairwise average nucleotide identity (ANI) was calculated using PYANI (32), and phylogenomic relationships were determined using PhyloPhlAn 3.0 with the following parameters: -d phylophlan -t a --diversity high --accurate -f supermatrix_aa.cfg (33). Genomic islands and regions of similarity were identified by reciprocal BLASTN and TBLASTX searches.

### Antimicrobial susceptibility testing

Susceptibility testing was performed against 24 antimicrobials spanning major classes: (i) **β-lactams**: amoxicillin, amoxicillin/clavulanic acid, piperacillin/tazobactam, cefuroxime, cefotaxime, ceftazidime, ceftazidime/avibactam, cefepime, ertapenem, imipenem, imipenem/relebactam, meropenem, meropenem/vaborbactam, aztreonam, aztreonam/avibactam, ceftolozane/tazobactam, and cefiderocol; (ii) **fluoroquinolones**: ciprofloxacin and delafloxacin; (iii) **aminoglycosides**: amikacin and tobramycin; and (iv) **others**: fosfomycin, tigecycline, and trimethoprim/sulfamethoxazole. Minimum inhibitory concentrations (MICs) were determined using E-test strips (bioMérieux) on Mueller–Hinton agar plates following EUCAST guidelines, incubated at 37°C for 24 h under aerobic conditions. Quality control strains *E. coli* ATCC 25922 and *P. aeruginosa* ATCC 27853 were included in each batch of tests. All E-test determinations were performed in duplicate to ensure reproducibility. Since no species-specific clinical breakpoints are currently available for *Shewanella* spp. in either EUCAST or CLSI guidelines, antimicrobial susceptibility results were interpreted according to the CLSI category Other Non-Enterobacterales (CLSI M100 ED35.2025), which is specifically intended for infrequently isolated, non-fermenting gram-negative bacilli not included in established taxonomic groups. This approach was selected as the most appropriate and conservative framework for the interpretation of susceptibility testing in an emerging, uncommon pathogen such as *S. algae*. Antimicrobial agents for which CLSI M100 (2025) Other Non-Enterobacterales breakpoints are available were interpreted categorically and reported as susceptibility rates. For agents lacking CLSI breakpoints, results were reported descriptively as MIC distributions only, without categorical interpretation.

### Resistance gene detection

Resistance determinants were screened from assembled genomes using the Comprehensive Antibiotic Resistance Database (CARD) (34) with thresholds of ≥70% identity and ≥50% coverage. This allowed determination of the prevalence and diversity of resistance determinants and correlation with phenotypic susceptibility patterns.

## RESULTS

### Phylogenomic diversity of *Shewanella algae*

We recovered a total of 86 *Shewanella algae* isolates from diverse sources, including 19 of clinical origin and 67 from environmental settings, and sequenced their genomes using Oxford Nanopore technology. To place these newly sequenced isolates (Table S1) within the broader genomic landscape of the species, we compared them with all publicly available *S. algae* genomes in NCBI (accessed February 2025). After quality filtering to exclude highly fragmented assemblies (>100 contigs), genomes with <90% completeness or >5% contamination, 178 public genomes were retained (Table S2; Fig. S1). These publicly available genomes used for comparison display a broad geographical distribution, predominantly from Asia but also from Europe, Africa, the Americas, and the Middle East, spanning multiple continents and ecological niches (Table S2; Fig. S1). Although the number of genomes remains modest relative to other bacterial species, this data set represents the current global diversity of *S. algae*.

**Fig 1:**
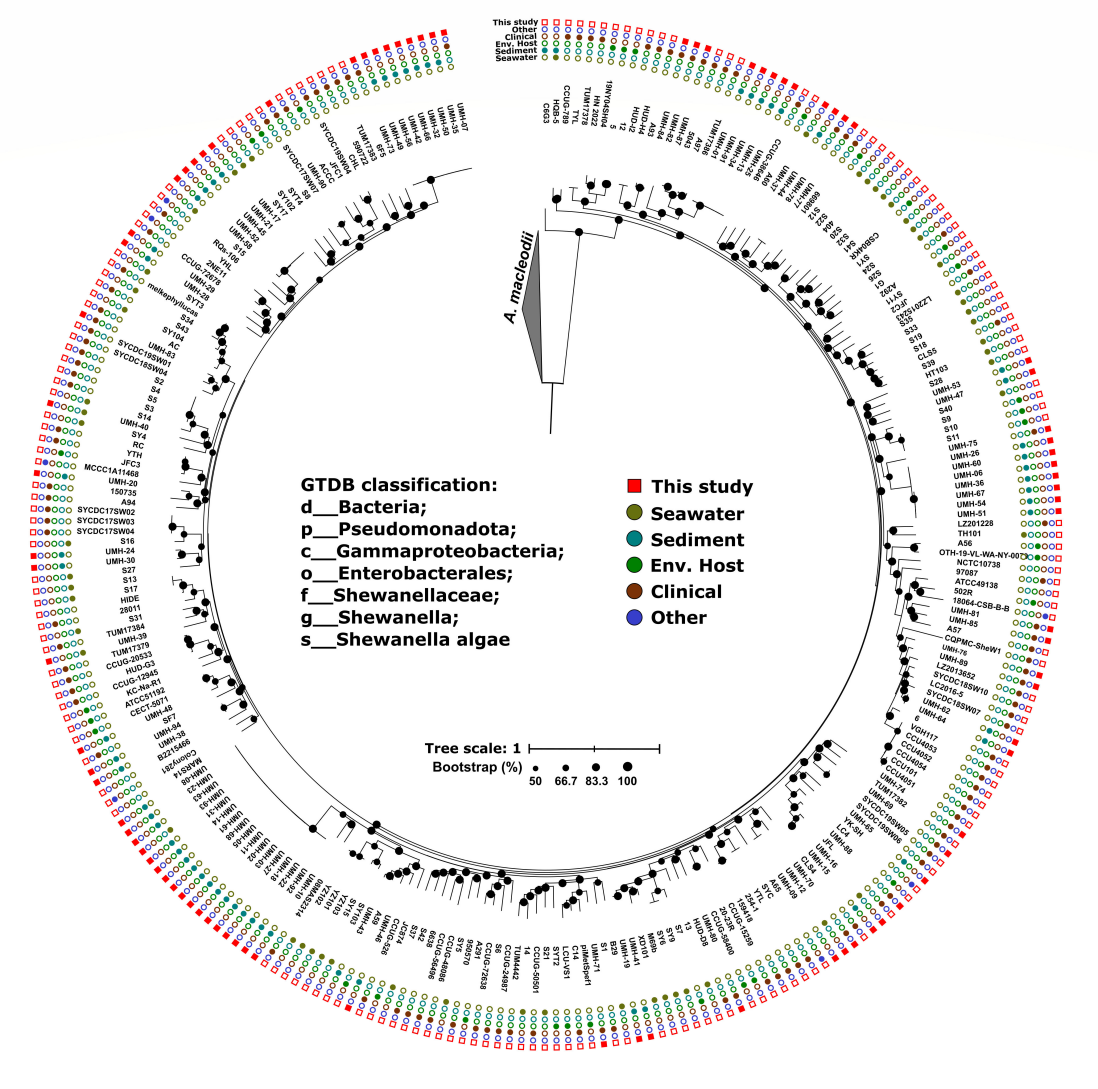
Maximum-likelihood phylogenomic tree of *Shewanella algae* genomes. The data set includes 178 publicly available genomes and 86 genomes sequenced in this study. Colored circles indicate the source of isolation, and squares mark newly sequenced isolates.

In total, 264 genomes (86 new and 178 public) were included in the phylogenomic analysis. A maximum-likelihood phylogenomic tree was constructed using a concatenated alignment of 152 universal single-copy marker proteins (Fig. 1). Genomes from *Alteromonas macleodii*, a closely related marine member of the class Gammaproteobacteria, were included as an outgroup to root the tree. The results showed that all new genomes were distributed throughout the phylogenetic tree, highlighting the broad local genomic heterogeneity of the species we obtained in our data set. Furthermore, analysis of average nucleotide identity (ANI) of the entire data set showed that the intrapopulation sequence diversity threshold for *S. algae* was 97.35%. This value is above the generally accepted species boundary of 95% ANI, suggesting relatively low genomic diversity within the species.

### Antimicrobial activity: MIC profiles and interpretative criteria

To complement the phylogenomic analysis, we next evaluated the *in vitro* activity of the antimicrobial agents tested against the 86 *S. algae* isolates. Minimum inhibitory concentration (MIC) measurements revealed marked differences in activity across the antibiotics evaluated (Table 1). The panel included agents commonly used in clinical practice, as well as recently introduced β-lactam/β-lactamase inhibitor combinations of emerging relevance. Among β-lactam agents, amoxicillin and amoxicillin/clavulanic acid showed limited activity, with high modal MICs (256 µg/mL) and elevated MIC_50_ and MIC_90_ values, indicating poor efficacy. In contrast, piperacillin/tazobactam (modal MIC = 0.25 µg/mL), cefuroxime (modal MIC = 1.5 µg/mL), and third- and fourth-generation cephalosporins (cefotaxime 0.094 µg/mL, ceftazidime 0.25 µg/mL, cefepime 0.064 µg/mL, ceftazidime/avibactam 0.094 µg/mL, and ceftolozane/tazobactam 0.125 µg/mL) all exhibited low modal MICs. Notably, the siderophore cephalosporin cefiderocol showed the lowest values (modal MIC = 0.016 µg/mL).

**TABLE 1:** Distribution of MIC_50_, MIC_90_, and modal MIC values (µg/mL) for antibiotics tested against *Shewanella algae* isolates (µg/mL) using E-test methodology^*a*^

Drug class		Antibiotic	MIC_50_	MIC_90_	Modal MIC
β-Lactams	Penicillins	Amoxicillin	64	256	256
		Amoxicillin/clavulanic acid*	48	256	256
		Piperacillin/tazobactam*	0.38	1	0.25
	Second-generation cephalosporin	Cefuroxime	2	4	1.5
	Third-generation cephalosporin	Cefotaxime	0.125	0.38	0.094
		Ceftazidime	0.25	0.75	0.25
		Ceftazidime/avibactam*	0.125	0.25	0.094
	Fourth-generation cephalosporin	Cefepime	0.047	0.094	0.064
		Ceftolozane/tazobactam*	0.125	0.19	0.125
	Siderophore cephalosporin	Cefiderocol	0.016	0.032	0.016
	Carbapenems	Ertapenem	0.125	0.5	0.25
		Imipenem	6	32	8
		Imipenem/relebactam*	6	32	6
		Meropenem	0.19	0.5	0.064
		Meropenem/vaborbactam*	0.25	1	0.38
	Monobactams	Aztreonam	0.38	0.75	0.38
		Aztreonam/avibactam*	0.25	0.38	0.25
Fluoroquinolones		Ciprofloxacin	0.125	0.19	0.125
		Delafloxacin	0.75	1.5	0.75
Aminoglycosides		Amikacin	3	4	3
		Tobramycin	1	1.5	1
Others		Fosfomycin	512	512	512
		Tigecycline	1	1.5	1
		Trimethoprim/sulfamethoxazole	0.125	0.19	0.125

^*a*^ Antibiotics marked with an asterisk (*) are combined with a β-lactamase inhibitor.

Within the carbapenem group, imipenem and imipenem/relebactam exhibited markedly higher MICs (modal MICs of 8 and 6 µg/mL, respectively) compared with ertapenem (0.25 µg/mL) and meropenem (0.064 µg/mL), highlighting an unusual discrepancy within the carbapenem group (Table 1). Meropenem/vaborbactam (0.38 µg/mL) retained excellent activity, similar to meropenem alone. Among fluoroquinolones, ciprofloxacin exhibited the greatest potency (0.125 µg/mL), whereas delafloxacin displayed reduced activity (0.75 µg/mL). For aminoglycosides, tobramycin achieved lower modal MICs (1 µg/mL) than amikacin (3 µg/mL), indicating superior *in vitro* activity (Table 1). Conversely, fosfomycin (512 µg/mL) exhibited very high MICs, consistent with poor efficacy. Tigecycline (1 µg/mL) showed borderline MICs, which may indicate reduced but not absent activity. By contrast, trimethoprim/sulfamethoxazole maintained uniformly low MICs (0.125 µg/mL), confirming robust activity against the isolate collection (Table 1).

Although a broad range of antibiotics was tested by E-test, susceptibility analysis was restricted to those agents for which CLSI provides breakpoints under the “Other Non-Enterobacterales” (CLSI M100 ED35.2025). When clinical (*n* = 19) and environmental (*n* = 67) isolates were analyzed separately, both groups displayed overall similar susceptibility patterns, with only minor differences for selected agents (Table 2). Among β-lactam agents, piperacillin/tazobactam showed high susceptibility rates (89.5% in clinical isolates and 100% in environmental isolates), and third- and fourth-generation cephalosporins (cefotaxime, ceftazidime, and cefepime) exhibited uniformly high values (>98% in both groups). Aztreonam showed greater variability, with lower activity among clinical isolates (78.9%) than among environmental ones (100%). Within carbapenems, imipenem displayed the lowest susceptibility (36.8% in clinical and 41.8% in environmental isolates), clearly diverging from meropenem, which showed markedly higher rates (89.5% and 100%, respectively). Among aminoglycosides, both amikacin and tobramycin exhibited high susceptibility (>94% in both groups). Ciprofloxacin also showed consistent activity (94.7% and 100%). Finally, trimethoprim/sulfamethoxazole demonstrated high susceptibility in both clinical and environmental isolates (94.7% and 100%) (Table 2).

**TABLE 2:** Susceptibility rates (%S) of clinical and environmental *Shewanella algae* isolates according to CLSI M100 ED35 (2025) “Other Non-Enterobacterales” criteria^*a*^

Antimicrobial agent		Clinical isolates (*n* = 19)	Environmental isolates (*n* = 67)
		%S	%S
β-Lactam combination agents	Piperacillin/tazobactam	89.5	100
Cephems	Cefotaxime	100	98.5
	Ceftazidime	100	100
	Cefepime	100	100
Monobactams	Aztreonam	78.9	100
Carbapenems	Imipenem	36.8	41.8
	Meropenem	89.5	100
Aminoglycosides	Amikacin	94.7	98.5
	Tobramycin	100	98.5
Fluoroquinolones	Ciprofloxacin	94.7	100
Folate pathway antagonists	Trimethoprim/sulfamethoxazole	94.7	100

^*a*^ S, susceptibility according to CLSI M100 ED35 (2025) “Other Non-Enterobacterales” criteria.

Overall, the close similarity between clinical and environmental isolates highlights the potential for environmental strains to act as reservoirs of resistance traits and reinforces the need for species-specific interpretive criteria. In the absence of *Shewanella*-specific breakpoints, the interpretation of antimicrobial susceptibility data remains constrained by the use of surrogate criteria, underscoring the importance of future studies aimed at defining standardized susceptibility thresholds for this emerging pathogen.

### Antibiotic resistance gene landscape

To complement the phenotypic susceptibility profiles, we analyzed the genomic basis of resistance using whole-genome sequencing. This approach enabled identification of both the intrinsic resistome, shared by nearly all isolates, and accessory resistance determinants potentially acquired by horizontal gene transfer (HGT).

**Fig 2:**
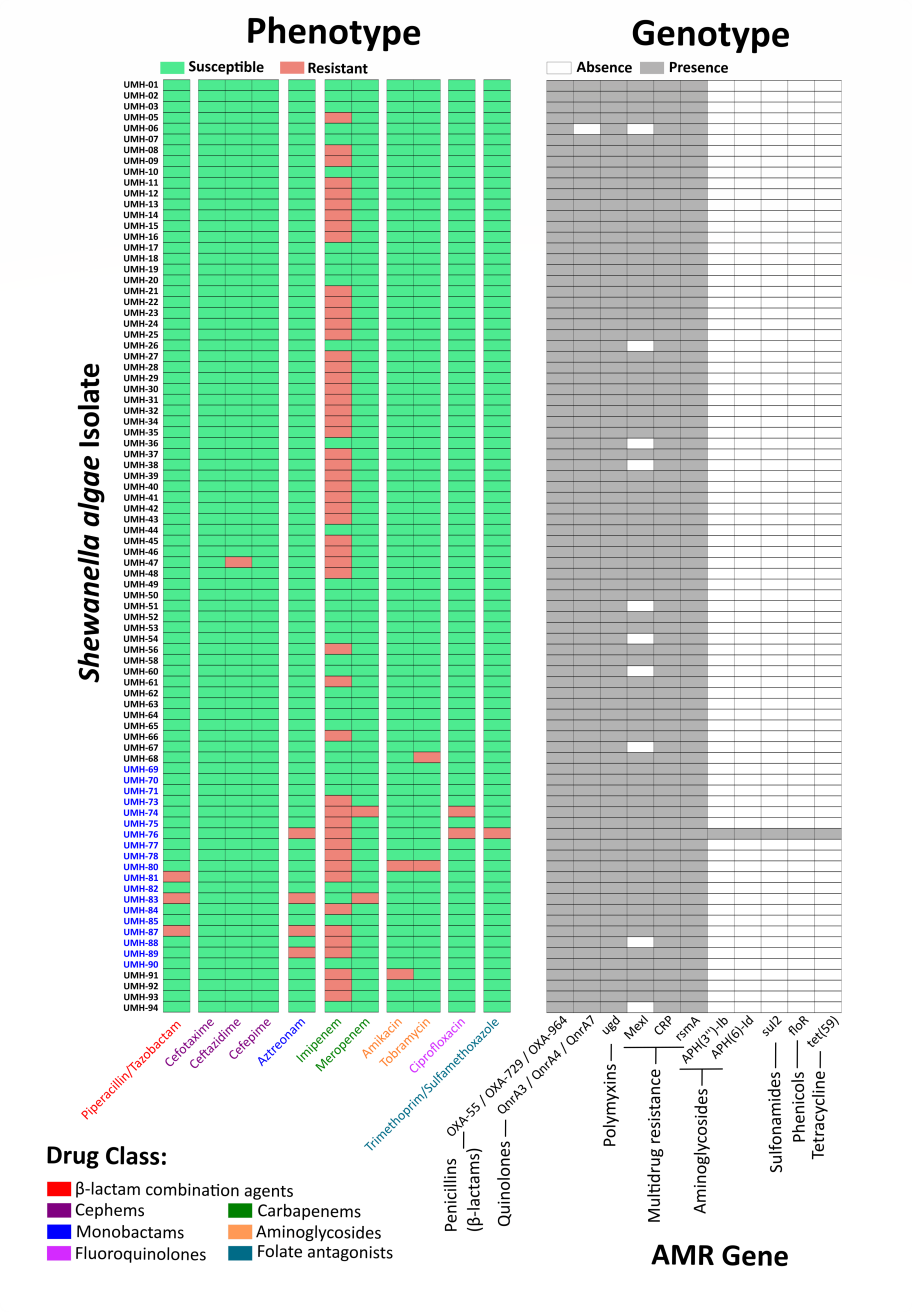
Correlation between phenotypic antimicrobial susceptibility profiles (interpreted using CLSI’s Other Non-Enterobacterales breakpoints) and the distribution of resistance genes in *Shewanella algae* isolates. Clinical strains are highlighted in blue, and environmental strains are highlighted in black.

Using the CARD database, the local core resistome (present in ≥95% of isolates) included β-lactamases (OXA-55, OXA-729, and OXA-964), quinolone resistance genes (*qnrA3*, *qnrA4*, and *qnrA7*), and *ugd*, a polymyxin resistance-associated gene occasionally detected in multiple copies (Fig. 2). These elements explain the baseline reduced susceptibility to β-lactams, quinolones, and polymyxins observed in phenotypic assays. In agreement with these intrinsic β-lactamases, our MIC data showed reduced activity of penicillins (amoxicillin and amoxicillin/clavulanic acid) and lower susceptibility to piperacillin/tazobactam, as well as a diminished intrinsic activity of imipenem, supporting a clear concordance between these genotypic determinants and the observed phenotypic resistance patterns. In addition, three efflux-related or regulatory genes were consistently found in the core resistome: *mexI* (MexGHI-OpmD multidrug efflux system), *crp* (repressor of *mdt*EF efflux), and *rsmA* (a global post-transcriptional regulator). The consistent presence of these efflux-related genes suggests an additional mechanism that may modulate intrinsic resistance to multiple antibiotic families, contributing to the phenotypic variability observed in some classes. Downregulation of *rsmA* has been associated with MexEF-OprN overexpression in other species, contributing to multidrug resistance phenotypes (35) (Fig. 2).

Beyond the conserved resistome, a particularly relevant finding was identified in the clinical isolate UMH-76, which carried five additional resistance genes embedded in a 29-kb genomic island. This island was flanked by an integrase, encoded P-type conjugation machinery (similar to the *tra* operon), and contained multiple IS91 family transposases, suggesting potential mobility and horizontal transfer. The resistance determinants included *APH(3″)-Ib* and *APH(6)-Id* (aminoglycosides), *sul2* (sulfonamides), *floR* (phenicols), and *tet(59*) (tetracyclines) (Fig. 2). Three additional genes with sequence identities below the predefined cutoff (70%), *tetR* (tetracycline resistance), *DfrA36* (trimethoprim resistance), and *NmcR* (β-lactam regulation) were also identified within this island. The latter is particularly relevant because *NmcR* regulates the *nmcA* carbapenemase in *Enterobacter cloacae* (36), raising the possibility of a similar regulatory mechanism in *S. algae*.

These findings indicate that *S. algae* harbors a dual resistance architecture: (i) a conserved intrinsic resistome accounting for baseline resistance phenotypes and (ii) accessory genomic elements, such as the 29-kb genomic island of the UMH-76 isolate, which expand the resistome and may enhance clinical adaptation.

### Distribution and mobility of the resistance island

**Fig 3:**
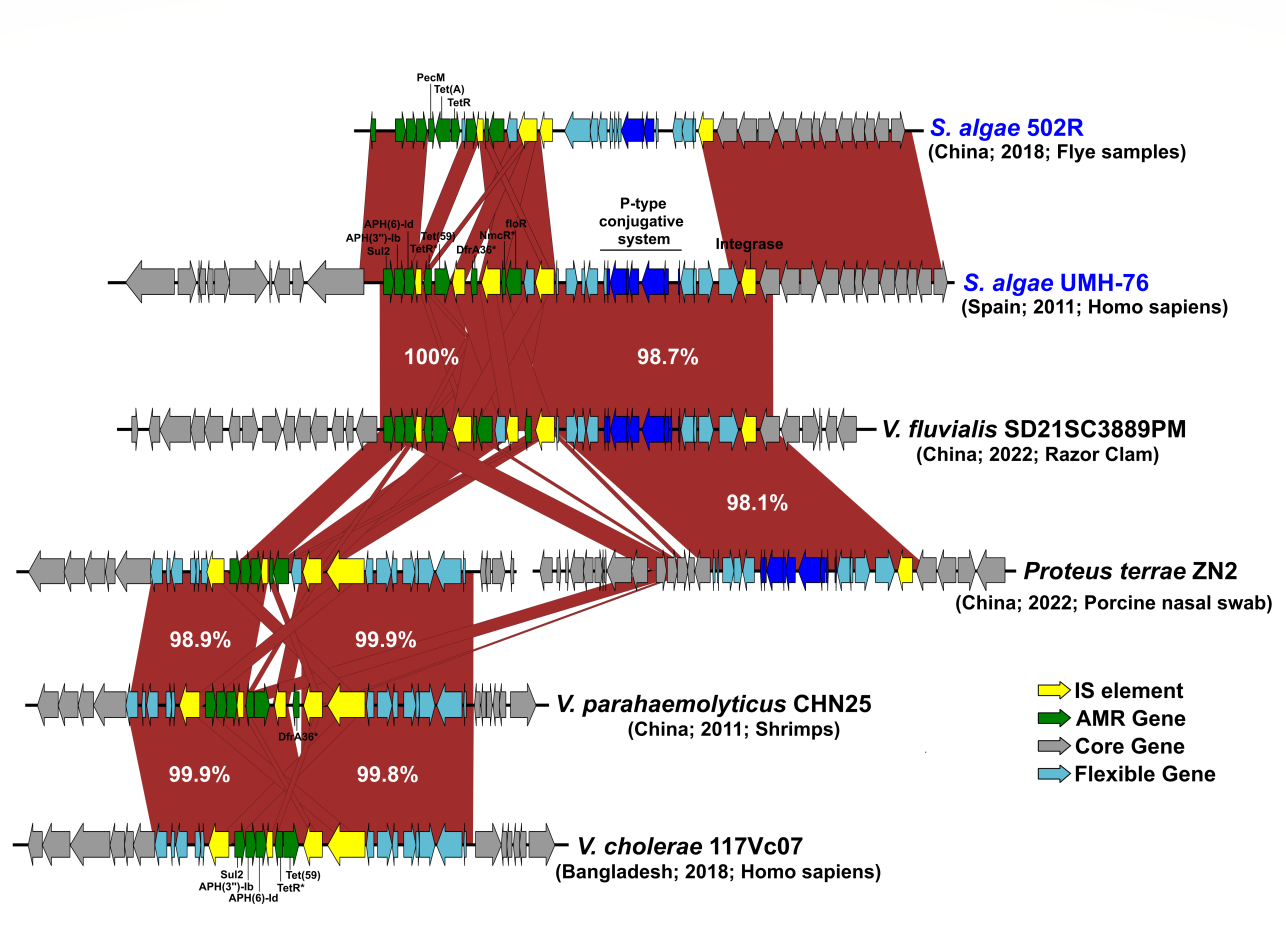
Comparative genomic alignment of the 29 kb resistance island from *Shewanella algae* strain UMH-76 with homologous regions identified within gammaproteobacteria.

To assess whether the 29-kb genomic island identified in the isolate UMH-76 represented a local event or a broader transferable platform, we examined its distribution across related genomes. BLAST analysis revealed a homologous island in *S. algae* strain 502R (30), inserted at the same genomic locus and flanked by a shared integrase, suggesting a hotspot for resistance module acquisition (Fig. 3). Although divergent, this island also carried conjugation genes and insertion sequence (IS)-associated resistance determinants. Compared to UMH-76, strain 502R lacks *dfrA36* and *tet(59*) but harbors *pecM*, *tet(A*), and *tetR* (Fig. 3).

The search for homologous regions also revealed highly similar structures in diverse Gammaproteobacteria, including three *Vibrio* species (*V. fluvialis*, *V. parahaemolyticus*, and *V. cholerae*) and *Proteus terrae*, suggesting horizontal gene transfer (HGT) events across both genera and families (Fig. 3). These species share similar ecological niches, particularly aquatic or humid environments, and are recognized as opportunistic human pathogens, underscoring the ecological overlap that may facilitate HGT. Of particular note, the same mobile genetic element was identified in *V. fluvialis* SD21SC3889PM and *P. terrae* ZN2, both isolated in China in 2022 but from distinct hosts, razor clam and swine, respectively. In *P. terrae* ZN2, the resistance island appeared split across two contigs, with one segment containing a P-type conjugation system and the other encompassing insertion sequences and resistance genes, highlighting the structural plasticity and potential mobility of this island (Fig. 3). Additionally, near-identical resistance clusters were detected in *V. parahaemolyticus* CHN25 (from shrimp, China) and *V. cholerae* 117Vc07 (from a human, Bangladesh), despite isolation differences in geography, host, and isolation year (8 years apart) (Fig. 3). These findings may indicate that the UMH-76 island is part of a shared, mobile resistance platform circulating among clinical and environmental Gammaproteobacteria. Its presence in both marine and terrestrial reservoirs underscores the potential for cross-species dissemination with significant clinical implications.

### Mobile integrons and plasmids as drivers of antimicrobial resistance spread in *Shewanella algae*

To define the core resistome, we initially analyzed the 86 locally sequenced isolates, for which both genotypic and phenotypic antimicrobial susceptibility data were available. This allowed us to establish a high-confidence baseline of resistance-associated genes. The same approach was then applied to the full data set of publicly available genomes to evaluate the consistency of the core resistome across the global *S. algae* population (Table S2). As expected, the intrinsic determinants identified in our isolates were consistently conserved across all genomes. However, 18 strains harbored additional resistance genes (Table S3). Among these, resistance determinants were located on plasmids in 11 genomes, within genomic islands in 6 genomes, and in a chromosomal integron in 1 genome (Table S3). Comparative analysis of these elements revealed that, irrespective of their genomic context, many shared modular structures flanked by insertion sequences (IS), facilitating mobility across strains. In most cases, these modules were associated with a class 1 integron–integrase (Fig. S2A). The presence of integrons in conjugative plasmids, previously described in major human pathogens (37), is referred to as a mobile integron. This type of mobile genetic element exhibits a dual mechanism of dissemination: conjugation genes (*tra*) enhance horizontal transfer, while integron promoters drive the expression of resistance genes.

A particularly illustrative case was *S. algae* strain KC-Na-R1 (Korea, 2017; GenBank GCA_003721455.1), isolated from *Neophocaena phocaenoides*, which carried plasmid pKC-Na-R1 (167 kb, GenBank CP033574.1). This plasmid was highly similar to one found in strain *S. algae* S19, recovered from seawater in China (2021, GenBank GCA_039615735.1) (Fig. S2A). Both plasmids contained a mobile integron with the same class 1 integrase, multiple IS-flanked resistance modules, and a complete mercury resistance operon, likely contributing to co-selection. Remarkably, pKC-Na-R1 encoded 16 resistance genes, some in multiple copies, conferring resistance to tetracyclines (tet[A]), rifamycins (arr-2 and arr-3), trimethoprim (dfrA27), sulfonamides (sul1), aminoglycosides (aadA16, ANT[3″]-IIa, ANT[2″]-Ia), fluoroquinolones (qnrA1), phenicols (cmlA5), and broad-spectrum β-lactams (OXA-10, VEB-1). Additionally, the presence of qacEΔ1, mediating resistance to quaternary ammonium compounds, reinforces the potential for co-selection. Nearly identical gene modules were also integrated into the chromosomes of *S. algae* strains 6 (human, China 2019) and S12 (*Chelonia mydas*; China, 2021) (Fig. S2A).

Further evidence came from *S. algae* strain 18064-65-CSB-B, which carried a plasmid encoding a class 1 integron–integrase and several resistance genes (*dfrA*, *qacEΔ1*, *sul1*, and *armA*) (p18064-65-CSB-B-B, 149 kb; GenBank CP047421.1). This plasmid conferred resistance to trimethoprim–sulfamethoxazole and aminoglycosides and reduced susceptibility to hospital disinfectants. BLAST searches revealed that homologous plasmids with high similarity (>98% id) circulate widely among Enterobacterales and *Vibrionaceae*, including clinical isolates (*Salmonella enterica* SAL-19-0623, Singapore, 2020; *Klebsiella pneumoniae* WRC02_S465MC, India, 2014; *Escherichia coli* M216, Myanmar, 2015; *Vibrio cholerae* M646, Bangladesh, 1979) as well as environmental or food-related strains (*Citrobacter werkmanii* BB1459 and *Citrobacter youngae* BB1468, Ghana, 2017; hospital wastewater; *Vibrio parahaemolyticus* Vb0499, shrimp isolate, China, 2015) (Fig. S2B).

The broad geographical and temporal distribution of these plasmids (1979–2020), coupled with their occurrence in both clinical and environmental contexts, highlights their role as highly versatile vehicles for disseminating resistance genes. These findings underscore the capacity of *S. algae* to act not only as an opportunistic pathogen but also as a reservoir and amplifier of mobile resistance determinants at the interface between aquatic environments and clinical settings.

## DISCUSSION

Human infections caused by *S. algae* are increasingly recognized as an emerging clinical concern, usually linked to aquatic exposures, particularly marine environments (22, 38). Once regarded as a purely environmental microorganism, *S. algae* has gained clinical relevance, with recent reports documenting a progressive rise in case numbers worldwide. Our study provides a comprehensive analysis of *S. algae* as both an opportunistic pathogen and a reservoir of antimicrobial resistance. A major strength is that it includes the largest set of environmental isolates reported to date, together with clinical strains from the same Mediterranean region, providing a representative snapshot of local diversity and potential routes of resistance transmission. The Mediterranean basin, where our isolates were collected, is undergoing rising sea surface temperatures, creating favorable conditions for the emergence of aquatic pathogens. Recent reports from France, Italy, and Spain describe cases of *S. algae* related to coastal exposure (13, 39, 40), paralleling the expansion of other marine pathogens such as *V. vulnificus* and *A. hydrophila* in temperate European waters (41–44). Our data suggest that *S. algae* may follow a similar trend in Europe, reinforcing the need for climate-informed surveillance. This underscores the need for clinical microbiology laboratories to be prepared for accurate detection and characterization of this species.

Taken together, the MIC data revealed a clear separation between antimicrobial agents with limited *in vitro* activity against *S. algae* and those that displayed marked potency. Penicillins and fosfomycin consistently showed high MIC values, indicating poor activity across the collection. In contrast, piperacillin/tazobactam, third- and fourth-generation cephalosporins, aminoglycosides, ciprofloxacin, trimethoprim–sulfamethoxazole, and several β-lactam/β-lactamase inhibitor combinations exhibited very low MIC_50_ and MIC_90_ values, reflecting strong *in vitro* activity. Recently introduced agents, including ceftazidime/avibactam, ceftolozane/tazobactam, and the siderophore cephalosporin cefiderocol, also demonstrated notable potency, highlighting their potential relevance for the treatment of *S. algae* infections. Carbapenems showed a particularly striking and atypical pattern. Imipenem and imipenem/relebactam displayed the highest MICs within the class, in marked contrast to meropenem and ertapenem, which showed substantially lower values. This intraclass discrepancy, uncommon among gram-negative non-fermenters, may reflect intrinsic permeability or regulatory traits specific to *S. algae* but also aligns with previous reports describing preferential hydrolysis of imipenem mediated by OXA-type β-lactamases intrinsically present in *Shewanella* spp., as well as potential species-specific differences in permeability and penicillin-binding proteins (45, 46). These intrinsic traits likely contribute to the distinct carbapenem susceptibility profile observed and further illustrate the limitations of extrapolating interpretive frameworks from other bacterial taxa.

When the data were interpreted according to CLSI’s “Other Non-Enterobacterales” criteria, clinical and environmental isolates exhibited largely overlapping susceptibility profiles. This close similarity supports the concept that environmental *S. algae* populations may act as reservoirs of phenotypic traits relevant to human infection and reinforces the need for species-specific interpretive criteria. In this context, imipenem emerged as the main agent for which susceptibility rates were markedly reduced, in contrast to meropenem and most other agents, underscoring the clinical uncertainty generated by the absence of dedicated breakpoints for this species.

Genomic analysis confirmed that our collection captures nearly the full diversity of the species, offering insights into resistance pathways. The intrinsic resistome, including OXA-type β-lactamases and *ugd*, explained baseline resistance to β-lactams and polymyxins, while accessory elements such as class 1 mobile integrons and genomic islands demonstrated the capacity of *S. algae* to acquire and disseminate resistance genes. Although phenotype–genotype correlations were generally consistent, discrepancies highlight current limitations. For instance, most of the strains exhibited phenotypic resistance to fosfomycin, but genomic analysis identified only a sequence with 30% identity to FosA, insufficient to confirm it as a functional homolog. This suggests alternative mechanisms, such as mutations in the target enzyme MurA, alterations in fosfomycin transporters GlpT or UhpT, or a previously uncharacterized FosA-like enzyme. Such findings emphasize that resistance gene databases are limited to known sequences, highlighting the need to complement genomic analyses with functional studies to accurately determine resistance mechanisms in *S. algae*. Although *qnrA* is also part of that core resistome and probably represents a species-associated determinant, its classification as intrinsic remains a matter of debate, as ancestral variants of *qnrA* from *Shewanella* have historically given rise to plasmid-mediated mobilized *qnr* genes in Enterobacterales (47). It is therefore acknowledged that this nuance must be taken into account when referring to qnrA within the core resistome.

*Shewanella* spp. have been recognized as ancestral reservoirs of carbapenemase genes that have subsequently disseminated to Enterobacterales. Several studies have documented the mobilization of bla-OXA-48-like and bla-NDM from *Shewanella* into clinically relevant species such as *Klebsiella pneumoniae* and *Escherichia coli* (18, 45, 48, 49). Consistent with these prior reports, our analysis showed that mobile integrons and plasmids emerged as major drivers of resistance gene dissemination, enabling HGT across distant taxa. Our identification of class 1 integron-associated modules in *S. algae*, with homologous structures in Enterobacterales and *Vibrionaceae*, further supports its role as a genetic intermediary at the land–sea–human interface. These findings emphasize that *S. algae* is not only an opportunistic pathogen but also a reservoir and amplifier of antimicrobial resistance genes with direct implications for global public health.

Importantly, we also found homologous resistance islands in *Vibrio* and *Proteus* species, highlighting the circulation of shared mobile elements across ecological niches. Because these organisms co-exist in aquatic and humid environments and include recognized human pathogens, this underscores the danger of environmental reservoirs as sources of clinically relevant resistance. The inclusion of a large number of environmental isolates in our study provides unique evidence of this ecological overlap, demonstrating that surveillance must extend beyond hospitals to environmental compartments.

Given the ecological distribution of *S. algae* at the interface of humans, animals, and the environment, a One Health perspective is essential. Surveillance strategies should integrate clinical, veterinary, and environmental sources to monitor resistance dissemination more effectively. Limitations of this study include that all MICs were determined using gradient diffusion (E-test) rather than the reference broth microdilution method, as well as sampling restricted to a single region and the absence of functional validation for predicted resistance genes. As noted by EUCAST, non-reference methods may be less reliable for certain antimicrobial agents. Therefore, categorical susceptibility interpretations, particularly in the absence of species-specific breakpoints, should be interpreted with caution. Future work should prioritize multicenter and longitudinal collections, experimental confirmation of integron activity, and investigations into cross-species transfer of mobile elements.

### Conclusion

In summary, our study demonstrates that *S. algae* is an emerging pathogen with significant clinical and ecological relevance. Local isolates spanned the global genomic diversity of the species and revealed a dual resistance architecture, combining intrinsic resistance genes with mobile genetic elements that enable HGT across bacterial taxa. These features highlight the dual role of *S. algae*: as a cause of human infections and as a reservoir of resistance determinants with potential for interspecies dissemination. Given its increasing incidence in southern Europe, the absence of species-specific breakpoints, and the identification of mobile resistance platforms, integrated surveillance strategies, future functional validation studies, and the establishment of tailored interpretive criteria are urgently needed to improve clinical management and mitigate its contribution to the spread of antimicrobial resistance.

## Data Availability

The genomes have been deposited in NCBI under BioProject accession number PRJNA1330513. Genome sequences are also publicly available in Zenodo (https://doi.org/10.5281/zenodo.18832680).

## SUPPLEMENTAL MATERIAL

Supplemental material aac.01891-25-s0001.pdf Fig. S1 and S2; Tables S1 to S3.

ASM does not own the copyrights to Supplemental Material that may be linked to, or accessed through, an article. The authors have granted ASM a non-exclusive, world-wide license to publish the Supplemental Material files. Please contact the corresponding author directly for reuse.
