# Supplementary material for: Bridging clinical and environmental reservoirs: antimicrobial resistance in the emerging pathogen Shewanella algae
Source: Antimicrob Agents Chemother. 2026 Apr 15;70(5):e01891-25. doi: 10.1128/aac.01891-25 (PMC13148052; doi:10.1128/aac.01891-25)
Supplement: Supplemental material — Fig. S1 and S2; Tables S1 to S3. [file aac.01891-25-s0001.pdf]

Bridging clinical and environmental reservoirs: Antimicrobial resistance in the emerging pathogen *Shewanella algae*

Celia García-Rivera[a], Juan J Roda-Garcia[b], Juan Carlos Rodríguez[a], Carmen Molina-Pardines[b], Iryna Tyshkovsca[a], Jose M Haro-Moreno[b], Antonio Martínez-Murcia[b,c], Maria Paz Ventero[a#], Mario López-Pérez[b#]

[a]Microbiology Department, Alicante University General Hospital, Alicante Institute of Sanitary and Biomedical Research (ISABIAL), Alicante, Spain; [b]Microbial Genomics and Evolution Group, División de Microbiología, Universidad Miguel Hernández, Alicante, Spain; [c]Genetic PCR Solutions®, Orihuela, Spain

[#]Corresponding authors: Mario López-Pérez (mario.lopezp@umh.es) and Maria Paz Ventero (maripazvm@gmail.com)

Supplementary Material

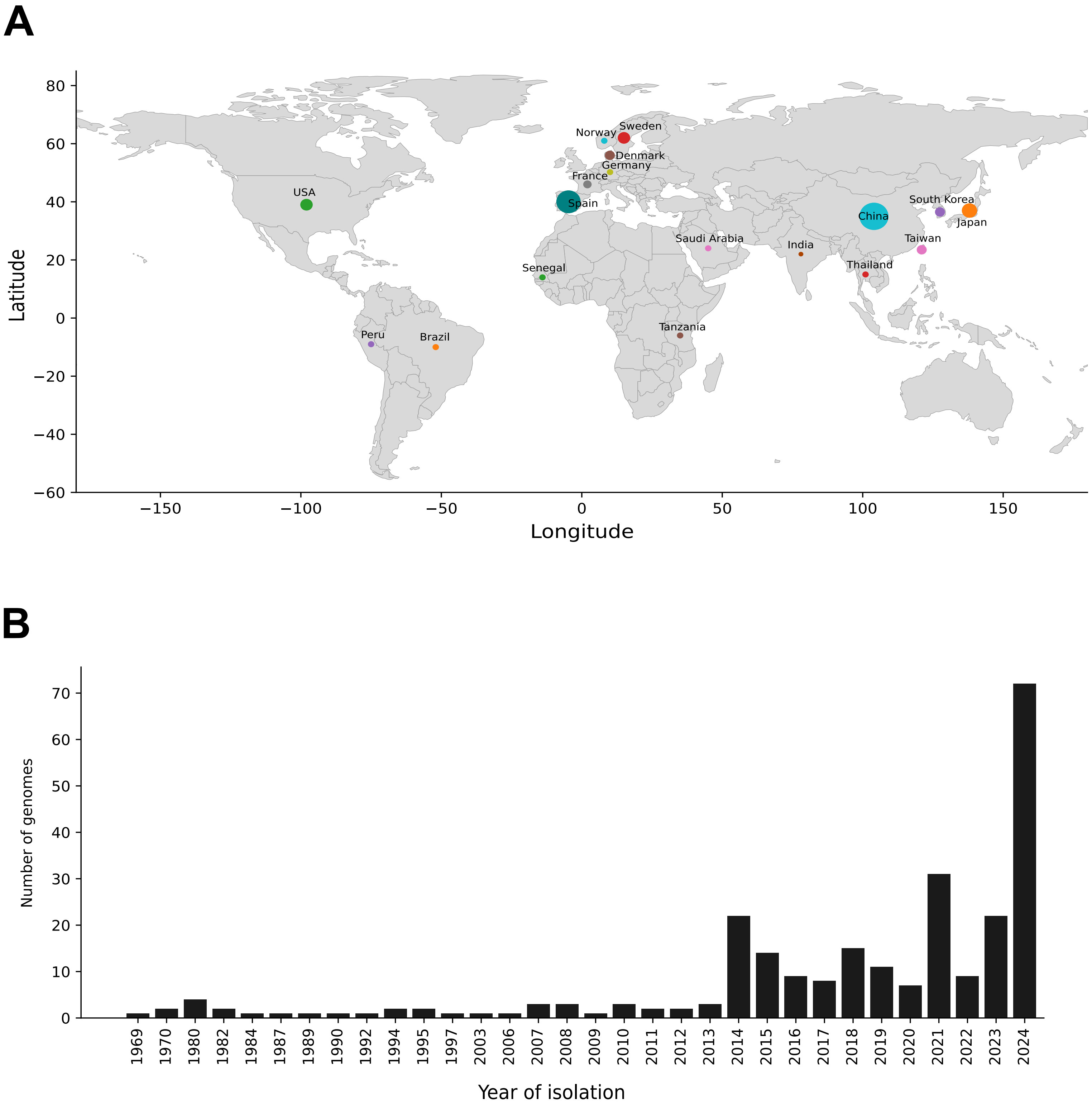

Figure S1. Geographic and temporal distribution of publicly available *Shewanella algae* genomes included in this study. A) Country of origin of genomes retrieved from NCBI. Genomes lacking geographic metadata were excluded. B) Histogram showing the number of genomes by year of isolation or release based on available metadata.

FIGURE-S2

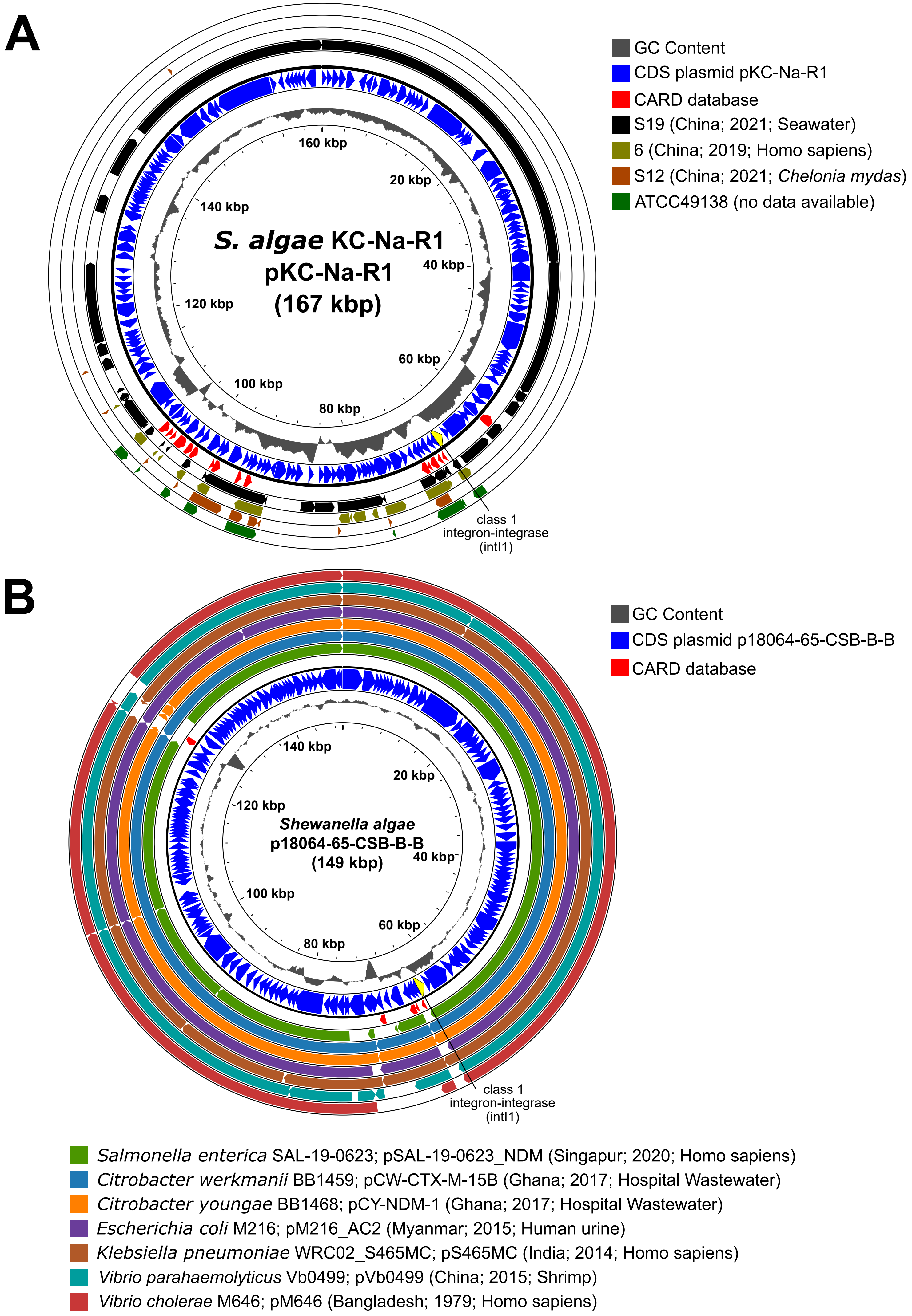
A
S. algae KC-Na-R1
pKC-Na-R1
(167 kbp)
160 kbp
20 kbp
40 kbp
60 kbp
80 kbp
100 kbp
120 kbp
140 kbp
class 1
integron-integrase
(intI1)
GC Content
CDS plasmid pKC-Na-R1
CARD database
S19 (China; 2021; Seawater)
6 (China; 2019; Homo sapiens)
S12 (China; 2021; Chelonia mydas)
ATCC49138 (no data available)
B
Shewanella algae
p18064-65-CSB-B-B
(149 kbp)
20 kbp
40 kbp
60 kbp
80 kbp
100 kbp
120 kbp
140 kbp
class 1
integron-integrase
(intI1)
GC Content
CDS plasmid p18064-65-CSB-B-B
CARD database
Salmonella enterica SAL-19-0623; pSAL-19-0623_NDM (Singapur; 2020; Homo sapiens)
Citrobacter werkmanii BB1459; pCW-CTX-M-15B (Ghana; 2017; Hospital Wastewater)
Citrobacter youngae BB1468; pCY-NDM-1 (Ghana; 2017; Hospital Wastewater)
Escherichia coli M216; pM216_AC2 (Myanmar; 2015; Human urine)
Klebsiella pneumoniae WRC02_S465MC; pS465MC (India; 2014; Homo sapiens)
Vibrio parahaemolyticus Vb0499; pVb0499 (China; 2015; Shrimp)
Vibrio cholerae M646; pM646 (Bangladesh; 1979; Homo sapiens)

Figure S2. A) Circular representations of the *Shewanella algae* plasmid pKC-Na-R1. Rings from the outside to inside: Circle 1. GC content. Circle 2. CDS of the pKC-Na-R1 (Blue). Circle 3. Determination of resistance genes using the CARD database (Red). Circles 4-7. Gene similarity in the genome of *S. algae* strains S19, 6, S12, and ATCC49138, respectively B). Circular representations of the *Shewanella algae* plasmid p18064-65-CSB-B-B. Rings from the outside to inside: Circle 1. GC content. Circle 2. CDS of the p18064-65-CSB-B-B (Blue). Circle 3. Determination of resistance genes using the CARD database (Red). Circles 4-7. Gene similarity found in plasmids from *Salmonella enterica* SAL-19-0623, *Citrobacter werkmanii* BB1459, *Citrobacter youngae* BB1468, *Escherichia coli* M216, *Klebsiella pneumoniae* WRC02_S465MC, *Vibrio parahaemolyticus* Vb0499, *Vibrio cholerae* M646, respectively.

Table S1. List of *Shewanella algae* collected in this study, together with genomic features and metadata

Organism Name	Strain	#Contigs	Genome length (bp)	GC content (%)	# of protein coding genes	Location	Year of Isolation	Source
Shewanella algae	UMH-01	40	4,831,851	53	4,359	Spain: Alicante	2024	Sediment
Shewanella algae	UMH-02	1	4,887,825	53	4,272	Spain: Alicante	2024	Sediment
Shewanella algae	UMH-03	1	4,887,827	53	4,273	Spain: Alicante	2024	Sediment
Shewanella algae	UMH-05	1	4,887,880	53	4,274	Spain: Alicante	2024	Sediment
Shewanella algae	UMH-06	1	5,049,309	53	4,450	Spain:alicante	2024	Sediment
Shewanella algae	UMH-07	1	5,000,929	53	4,369	Spain: Alicante	2024	Sediment
Shewanella algae	UMH-08	1	4,887,877	53	4,272	Spain: Alicante	2024	Seawater
Shewanella algae	UMH-09	1	4,851,958	53	4,237	Spain: Alicante	2024	Sediment
Shewanella algae	UMH-10	2	4,978,892	53	4,394	Spain: Alicante	2024	Sediment
Shewanella algae	UMH-11	1	4,889,435	53	4,278	Spain: Alicante	2024	Sediment
Shewanella algae	UMH-12	1	4,851,958	53	4,239	Spain: Alicante	2024	Seawater
Shewanella algae	UMH-13	1	4,998,731	53	4,349	Spain: Alicante	2024	Sediment
Shewanella algae	UMH-14	1	4,889,322	53	4,276	Spain: Alicante	2024	Sediment
Shewanella algae	UMH-15	1	4,947,277	53	4,371	Spain: Alicante	2024	Sediment
Shewanella algae	UMH-16	1	4,947,256	53	4,362	Spain: Alicante	2024	Seawater
Shewanella algae	UMH-17	1	4,915,548	53	4,283	Spain: Alicante	2024	Seawater
Shewanella algae	UMH-18	2	4,947,772	53	4,347	Spain: Alicante	2024	Sediment
Shewanella algae	UMH-19	1	4,951,923	53	4,365	Spain: Alicante	2024	Sediment
Shewanella algae	UMH-20	1	4,904,624	53	4,266	Spain: Alicante	2024	Sediment
Shewanella algae	UMH-21	1	4,915,278	53	4,284	Spain: Alicante	2024	Sediment
Shewanella algae	UMH-22	3	5,036,898	53	4,463	Spain: Alicante	2024	Sediment
Shewanella algae	UMH-23	1	4,887,871	53	4,276	Spain: Alicante	2024	Sediment
Shewanella algae	UMH-24	1	4,950,587	53	4,316	Spain: Alicante	2024	Sediment
Shewanella algae	UMH-25	1	4,998,735	53	4,348	Spain: Alicante	2024	Sediment
Shewanella algae	UMH-26	3	5,021,699	53	4,416	Spain:alicante	2024	Sediment
Shewanella algae	UMH-27	1	4,887,826	53	4,273	Spain: Alicante	2024	Sediment
Shewanella algae	UMH-28	1	4,961,287	53	4,354	Spain: Alicante	2024	Sediment
Shewanella algae	UMH-29	1	4,945,522	53	4,323	Spain: Alicante	2024	Sediment
Shewanella algae	UMH-30	1	4,933,750	53	4,314	Spain: Alicante	2024	Sediment
Shewanella algae	UMH-31	1	4,889,323	53	4,274	Spain: Alicante	2024	Sediment
Shewanella algae	UMH-32	1	5,000,928	53	4,368	Spain: Alicante	2024	Sediment
Shewanella algae	UMH-34	1	4,998,206	53	4,351	Spain: Alicante	2024	Sediment
Shewanella algae	UMH-35	1	5,011,827	53	4,385	Spain: Alicante	2024	Sediment
Shewanella algae	UMH-36	1	5,049,309	53	4,453	Spain:alicante	2024	Sediment
Shewanella algae	UMH-37	3	4,986,824	53	4,403	Spain: Alicante	2024	Seawater
Shewanella algae	UMH-38	1	5,027,716	53	4,452	Spain: Alicante	2024	Sediment
Shewanella algae	UMH-39	2	4,979,979	53	4,437	Spain: Alicante	2024	Sediment
Shewanella algae	UMH-40	1	4,989,419	53	4,368	Spain: Alicante	2024	Seawater
Shewanella algae	UMH-41	2	5,032,097	53	4,466	Spain: Alicante	2024	Sediment
Shewanella algae	UMH-42	1	5,007,479	53	4,377	Spain: Alicante	2024	Sediment
Shewanella algae	UMH-43	1	4,842,022	53	4,233	Spain: Alicante	2024	Sediment
Shewanella algae	UMH-44	3	5,037,061	53	4,431	Spain: Alicante	2024	Sediment
Shewanella algae	UMH-45	1	4,915,563	53	4,284	Spain: Alicante	2024	Sediment
Shewanella algae	UMH-46	1	4,839,523	53	4,213	Spain: Alicante	2024	Sediment
Shewanella algae	UMH-47	1	5,002,780	53	4,380	Spain: Alicante	2024	Sediment
Shewanella algae	UMH-48	1	5,091,532	53	4,530	Spain: Alicante	2024	Sediment
Shewanella algae	UMH-49	1	5,006,030	53	4,377	Spain: Alicante	2024	Sediment
Shewanella algae	UMH-50	1	4,967,503	53	4,324	Spain: Alicante	2024	Sediment
Shewanella algae	UMH-51	7	4,997,621	53	4,392	Spain:alicante	2024	Sediment
Shewanella algae	UMH-52	9	4,844,756	53	4,269	Spain: Alicante	2024	Sediment
Shewanella algae	UMH-53	1	4,896,065	53	4,313	Spain: Alicante	2024	Sediment
Shewanella algae	UMH-54	2	5,020,368	53	4,421	Spain:alicante	2024	Sediment
Shewanella algae	UMH-56	1	4,974,118	53	4,330	Spain: Alicante	2024	Sediment
Shewanella algae	UMH-58	52	5,349,527	53	4,722	Spain: Alicante	2024	Sediment
Shewanella algae	UMH-60	1	5,003,856	53	4,394	Spain:alicante	2024	Sediment
Shewanella algae	UMH-61	1	4,887,866	53	4,273	Spain: Alicante	2024	Sediment
Shewanella algae	UMH-62	4	4,882,395	53	4,376	Spain: Alicante	2024	Sediment
Shewanella algae	UMH-63	1	4,887,882	53	4,274	Spain: Alicante	2024	Sediment
Shewanella algae	UMH-64	2	4,888,252	53	4,394	Spain: Alicante	2024	Sediment
Shewanella algae	UMH-65	1	4,960,069	53	4,346	Spain: Alicante	2024	Sediment
Shewanella algae	UMH-66	1	4,974,117	53	4,330	Spain: Alicante	2024	Sediment
Shewanella algae	UMH-67	7	5,041,215	53	4,451	Spain:alicante	2024	Sediment
Shewanella algae	UMH-68	4	4,967,325	53	4,375	Spain: Alicante	2024	Sediment
Shewanella algae	UMH-69	1	4,929,853	53	4,332	Spain: Elche	2023	Homo sapiens
Shewanella algae	UMH-70	2	4,947,505	53	4,345	Spain: Alicante	2023	Homo sapiens
Shewanella algae	UMH-71	1	4,907,840	53	4,274	Spain: Santader	2006-2020	Homo sapiens
Shewanella algae	UMH-73	1	4,894,115	53	4,317	Spain: Santader	2006-2020	Homo sapiens
Shewanella algae	UMH-74	2	5,072,662	53	4,495	Spain: Santader	2006-2020	Homo sapiens
Shewanella algae	UMH-75	2	4,892,141	53	4,281	Spain: Santader	2006-2020	Homo sapiens
Shewanella algae	UMH-76	1	4,771,256	53	4,210	Spain: Santader	2006-2020	Homo sapiens
Shewanella algae	UMH-77	1	5,038,167	53	4,471	Spain: Santader	2006-2020	Homo sapiens
Shewanella algae	UMH-78	1	5,039,495	53	4,470	Spain: Santader	2006-2020	Homo sapiens
Shewanella algae	UMH-80	1	4,835,349	53	4,200	Spain: Santader	2006-2020	Homo sapiens
Shewanella algae	UMH-81	2	4,758,811	53	4,217	Spain: Santader	2006-2020	Homo sapiens
Shewanella algae	UMH-82	1	4,907,973	53	4,315	Spain: Santader	2006-2020	Homo sapiens
Shewanella algae	UMH-83	1	4,827,729	53	4,189	Spain: Santader	2006-2020	Homo sapiens
Shewanella algae	UMH-84	1	4,887,014	53	4,248	Spain: Santader	2006-2020	Homo sapiens
Shewanella algae	UMH-85	2	4,784,013	53	4,224	Spain: Santader	2023	Homo sapiens
Shewanella algae	UMH-87	1	5,047,050	53	4,490	Spain: Alicante	2024	Homo sapiens
Shewanella algae	UMH-88	1	4,892,412	53	4,287	Spain: Alicante	2024	Homo sapiens
Shewanella algae	UMH-89	1	4,816,125	53	4,262	Spain: Alicante	2024	Homo sapiens
Shewanella algae	UMH-90	2	4,919,849	53	4,355	Spain: Alicante	2023	Homo sapiens
Shewanella algae	UMH-91	1	4,982,176	53	4,331	Spain: Alicante	2024	Sediment
Shewanella algae	UMH-92	3	4,951,799	53	4,350	Spain: Alicante	2024	Sediment
Shewanella algae	UMH-93	3	4,895,273	53	4,281	Spain: Alicante	2024	Seawater
Shewanella algae	UMH-94	1	5,030,585	53	4,456	Spain: Alicante	2024	Sediment

Table S2. Publicly available *S. algae* genomes retrieved from NCBI, with associated genomic features and metadata.

Organism Name	Strain	#Contigs	Genome length (bp)	GC content (%)	Year of Isolation	Source	Location	Completeness (%)	Contamination (%)
Shewanella algae	08MAS2314	55	4,851,061	53	2008	Homo sapiens	China: Ma'anshan	100	0.5
Shewanella algae	12	46	4,828,472	53	2018	Delphinidae	China	100	0.1
Shewanella algae	13	44	4,917,425	53	2023	Seawater	China: sanya city	100	0.4
Shewanella algae	14	40	4,976,312	53	2019	Seawater	China	100	0.2
Shewanella algae	150735	1	5,070,545	53	2015	Homo sapiens	Spain:Gran Canaria	100	0.2
Shewanella algae	159418	62	4,769,540	53	2015	Homo sapiens	Spain:Gran Canaria	100	0.2
Shewanella algae	18064-CSB-B-B	2	4,903,874	53	2018	Poultry	Tanzania	100	0.2
Shewanella algae	19NY04SH04	2	5,048,574	53	2019	shrimp	USA	100	0.4
Shewanella algae	20-23R	56	4,758,780	53	N.D	Fish (Upeneus japonicus)	South Korea	100	0.3
Shewanella algae	254-1	62	4,763,835	53	2014	Homo sapiens	Spain:Gran Canaria	100	0.2
Shewanella algae	28011	71	4,943,711	53	2014	Homo sapiens	Spain:Gran Canaria	100	0.2
Shewanella algae	2NE11	1	5,030,813	53	2018	Olive oil	Peru	100	0.1
Shewanella algae	404	71	4,880,812	53	2021	Seawater	China	100	0.2
Shewanella algae	5	46	4,827,936	53	2018	Delphinidae	China	100	0.1
Shewanella algae	502R	89	4,715,342	53	2018	Flye samples	China: Henan	100	0.2
Shewanella algae	5043	52	4,997,180	53	2015	Homo sapiens	Spain: Gran Canaria	100	0.4
Shewanella algae	590722	74	4,992,475	53	2018	Homo sapiens	Spain:Gran Canaria	100	0.2
Shewanella algae	6	72	4,653,983	53	2019	Homo sapiens	China	100	0.1
Shewanella algae	6638	35	4,851,331	53	2019	Homo sapiens	China	100	1.1
Shewanella algae	669801	36	4,776,174	53	2016	Homo sapiens	Spain:Gran Canaria	100	0.5
Shewanella algae	6F5	71	4,870,277	53	2011	Homo sapiens	Sapin: Gran Canaria	100	0.2
Shewanella algae	950570	46	4,756,369	53	2019	Homo sapiens	Spain: Granada	100	0.1
Shewanella algae	97087	98	4,856,502	53	2015	Homo sapiens	Spain: Granada	100	0.2
Shewanella algae	A291	1	4,971,565	53	1995	fish	Denmark	100	0.2
Shewanella algae	A292	46	4,957,768	53	1995	fish	Denmark	100	0.1
Shewanella algae	A56	51	4,848,386	53	1980	Fish	Senegal	100	0.2
Shewanella algae	A57	95	4,660,999	53	1980	Homo sapiens	France	100	0.2
Shewanella algae	A59	1	4,817,651	53	1980	Flamingo	France	100	0.2
Shewanella algae	A60	38	4,818,514	53	1980	Flamingo	France	100	0.2
Shewanella algae	A65	61	4,901,782	53	1982	Poultry	France	100	0.6
Shewanella algae	A93	64	4,913,852	53	1994	Homo sapiens	Denmark	100	0.3
Shewanella algae	A94	44	4,882,077	53	1994	Homo sapiens	Denmark	100	0.1
Shewanella algae	A97	62	5,005,946	53	1992	Homo sapiens	Denmark	100	0.2
Shewanella algae	AC	22	4,723,144	53	N.D	Homo sapiens	Spain: Santader	99.99	0.2
Shewanella algae	ACCC	74	4,744,804	53	2014	Homo sapiens	Taiwan	100	0.2
Shewanella algae	ATCC49138	1	4,849,690	53	mising	mising	mising	100	0.3
Shewanella algae	ATCC51192	52	4,978,360	52	N.D	Red alga	France: Arcachon Bay	100	0.2
Shewanella algae	B2215466	1	4,946,841	53	2022	Homo sapiens	Germany	100	0.1
Shewanella algae	B29	88	4,831,396	53	2022	Seawater	Saudi Arabia	100	0.2
Shewanella algae	C14	79	4,846,373	53	2023	Homo sapiens	China	100	0.1
Shewanella algae	C6G3	43	4,879,425	53	2007	sediment	France: Arcahon Bay	88.74	3.4
Shewanella algae	CCU101	2	4,919,337	53	2013	Homo sapiens	Taiwan	100	0.2
Shewanella algae	CCU4051	2	4,919,537	53	2016	Homo sapiens	Taiwan	100	0.2
Shewanella algae	CCU4052	2	4,919,930	53	2016	Homo sapiens	Taiwan	100	0.2
Shewanella algae	CCU4053	2	4,919,868	53	2016	Homo sapiens	Taiwan	100	0.1
Shewanella algae	CCU4054	2	4,919,717	53	2016	Homo sapiens	Taiwan	99.99	0.4
Shewanella algae	CCUG-12945	40	4,875,886	53	1982	Homo sapiens	USA	100	0.1
Shewanella algae	CCUG-15259	50	4,891,496	53	1984	Homo sapiens	USA	100	0.3
Shewanella algae	CCUG-20533	57	4,784,483	53	1987	Homo sapiens	Sweden	100	0.1
Shewanella algae	CCUG-24987	89	4,814,300	53	1989	Homo sapiens	Sweden	100	0.2
Shewanella algae	CCUG-38646	35	4,790,201	53	1997	Homo sapiens	Norway	100	0.2
Shewanella algae	CCUG-48086	33	4,844,414	53	2003	Homo sapiens	Sweden	100	0.2
Shewanella algae	CCUG-50501	71	5,016,616	53	2010	Homo sapiens	Sweden	100	0.1
Shewanella algae	CCUG-526	45	4,784,369	53	1969	Homo sapiens	Sweden	100	0.2
Shewanella algae	CCUG-56496	38	4,796,178	53	2008	Homo sapiens	Sweden	100	0.1
Shewanella algae	CCUG-58400	51	4,766,857	53	2009	Fish (Upeneus japonicus)	South Korea	100	0.3
Shewanella algae	CCUG-72638	60	4,876,138	53	2018	Homo sapiens	Sweden	100	0.2
Shewanella algae	CCUG-72678	46	4,870,233	53	2018	Homo sapiens	Sweden	100	0.2
Shewanella algae	CCUG-789	62	4,863,341	53	1970	Homo sapiens	USA	100	0.3
Shewanella algae	CECT-5071	1	4,924,764	53	1990	Algae (Jania sp.)	Japan	100	0.2
Shewanella algae	CHL	55	4,888,589	53	2014	Homo sapiens	Taiwan	100	0.1
Shewanella algae	CLS4	35	4,866,748	53	2014	Homo sapiens	Taiwan	100	0.1
Shewanella algae	CLS5	43	4,824,095	53	2014	Homo sapiens	Taiwan	100	0.1
Shewanella algae	Colony281	1	4,990,025	53	N.D	Food outbreaks	Thailand	100	0.1
Shewanella algae	CQPMC-SheW1	1	4,891,834	53	2024	Homo sapiens	China	88.42	4.0
Shewanella algae	CSB04KR	64	4,803,356	53	2015	Apostichopus japonicus	South Korea	100	3.4
Shewanella algae	G1	1	4,887,746	53	2016	Homo sapiens	Spain:Granada	100	0.2
Shewanella algae	HIDE	1	4,950,784	53	2015	Homo sapiens	Taiwan	100	0.3
Shewanella algae	HN2022	57	4,797,750	53	2022	Homo sapiens	China	100	0.2
Shewanella algae	HQB-5	8	4,779,819	53	2017	Sediments	China	100	0.3
Shewanella algae	HT103	53	4,860,262	53	2023	Seawater	China	100	0.4
Shewanella algae	HUD-D8	36	4,831,989	53	2016	Homo sapiens	Sweden	100	0.2
Shewanella algae	HUD-G3	69	4,980,582	53	2018	Homo sapiens	Sweden	100	0.1
Shewanella algae	HUD-H4	60	4,932,276	53	2018	Homo sapiens	Sweden	100	0.1
Shewanella algae	HUD-I2	61	4,932,981	53	2018	Homo sapiens	Sweden	100	0.1
Shewanella algae	JC874	71	4,839,786	53	2022	Algae (Green algae)	India	100	0.3
Shewanella algae	JFC1	91	4,803,344	53	2014	Oysters (Magallana gigas)	Taiwan	100	0.1
Shewanella algae	JFC2	44	4,840,125	53	2014	Crassostrea gigas	Taiwan	100	0.2
Shewanella algae	JFC3	43	4,819,646	53	2014	Oyster (Crassostrea gigas)	Taiwan	100	0.2
Shewanella algae	JFL	41	4,801,112	53	2014	Homo sapiens	Taiwan	100	0.1
Shewanella algae	KC-Na-R1	2	5,203,444	53	2017	Cetaceans (Neophocaena)	South Korea	100	0.3
Shewanella algae	LC2016-5	82	4,940,147	53	2016	Homo sapiens	China: Laizhou	100	0.8
Shewanella algae	LC4	56	5,014,045	53	2015	Corals (Siderastrea stellata)	Brazil	100	8.5
Shewanella algae	LCU-VS1	88	4,794,921	53	2022	Shrimp (Penaeus vannamei)	China	100	0.1
Shewanella algae	LZ201228	33	4,939,117	53	2012	Homo sapiens	China: Laizhou	100	2.8
Shewanella algae	LZ2013652	95	4,709,042	53	2013	Homo sapiens	China: Laizhou	100	0.2
Shewanella algae	LZ2015243	89	4,803,486	53	2015	Homo sapiens	China: Laizhou	100	0.1
Shewanella algae	M69b	48	4,868,919	53	2021	Seawater	Saudi Arabia	100	0.2
Shewanella algae	MARS14	11	5,005,849	53	2015	N.D	N.D	100	0.1
Shewanella algae	MCCC1A11468	1	4,880,117	53	2017	Single cell	China	100	0.2
Shewanella algae	melkephyllucas	51	4,808,216	53	2014	Homo sapiens	Taiwan	100	0.3
Shewanella algae	NCTC10738	3	4,983,583	53	1970	N.D	N.D	100	0.1
Shewanella algae	OTH-19-VL-WA-NY-0079	75	4,961,640	53	2019	Fish (Canthigaster valentini)	USA	100	0.3
Shewanella algae	piMetSpef1	1	4,824,153	53	2021	Ciliates (Metopus)	USA	100	0.2
Shewanella algae	RC	54	4,807,239	53	2014	Homo sapiens	Taiwan	100	0.2
Shewanella algae	RQs-106	1	4,990,025	53	2011	activated sludge	China: Dalian	100	0.4
Shewanella algae	S1	66	4,847,794	53	2021	Homo sapiens	China	100	0.2
Shewanella algae	S10	73	4,868,218	53	2021	Eretmochelys imbricata	China	100	0.3
Shewanella algae	S11	73	4,866,492	53	2021	Eretmochelys imbricata	China	100	0.3
Shewanella algae	S12	51	5,035,735	53	2021	Chelonia mydas	China	100	0.1
Shewanella algae	S13	42	4,815,374	53	2020	Seawater	China	100	0.2
Shewanella algae	S14	42	4,764,266	53	2020	Seawater	China	100	0.1
Shewanella algae	S15	30	4,851,818	53	2020	Seawater	China	100	0.4
Shewanella algae	S16	77	4,788,612	53	2020	Seawater	China	100	0.2
Shewanella algae	S17	44	4,785,825	53	2020	Seawater	China	100	0.2
Shewanella algae	S18	47	4,818,909	53	2021	Seawater	China	100	0.2
Shewanella algae	S19	57	5,002,533	53	2021	Seawater	China	100	0.1
Shewanella algae	S2	51	4,839,075	53	2021	Homo sapiens	China	100	0.2
Shewanella algae	S20	68	4,913,113	53	2021	Seawater	China	100	0.2
Shewanella algae	S21	74	4,817,340	53	2021	Seawater	China	100	0.1
Shewanella algae	S22	40	4,923,813	53	2021	Seawater	China	100	0.1
Shewanella algae	S24	85	4,899,438	53	2021	Seawater	China	100	4.6
Shewanella algae	S26	99	4,872,336	53	2022	Seawater	China	100	0.9
Shewanella algae	S27	46	4,915,102	53	2021	Seawater	China	100	0.2
Shewanella algae	S28	33	4,851,549	53	2021	Seawater	China	100	0.2
Shewanella algae	S3	50	4,840,473	53	2022	Homo sapiens	China	100	0.2
Shewanella algae	S31	64	4,913,477	53	2021	Seawater	China	100	0.2
Shewanella algae	S32	45	4,785,735	53	2021	Seawater	China	100	0.2
Shewanella algae	S33	48	4,861,667	53	2021	Seawater	China	100	0.1
Shewanella algae	S34	37	4,852,408	53	2021	Seawater	China	100	0.3
Shewanella algae	S35	75	5,004,580	53	2021	Seawater	China	100	0.2
Shewanella algae	S37	34	4,969,485	53	2021	Seawater	China	100	2.1
Shewanella algae	S39	51	4,785,193	53	2021	Seawater	China	100	1.0
Shewanella algae	S4	50	4,837,844	53	2021	Homo sapiens	China	100	0.2
Shewanella algae	S40	53	5,069,133	53	2021	Seawater	China	100	4.9
Shewanella algae	S41	53	4,805,524	53	2022	Seawater	China	100	0.1
Shewanella algae	S42	56	4,904,141	53	2021	Homo sapiens	China	100	0.2
Shewanella algae	S43	36	4,807,422	53	2021	Homo sapiens	China	100	0.3
Shewanella algae	S5	52	4,839,325	53	2021	Homo sapiens	China	100	0.2
Shewanella algae	S6	70	4,870,429	53	2022	Homo sapiens	China	100	0.3
Shewanella algae	S7	21	4,917,381	53	2021	Eretmochelys imbricata	China	100	0.1
Shewanella algae	S8	49	4,968,144	53	2021	Turtle (Eretmochelys imbricata)	China	100	0.2
Shewanella algae	S9	78	4,866,853	53	2021	Eretmochelys imbricata	China	100	0.3
Shewanella algae	SF7	51	4,843,605	53	2010	Homo sapiens	Spain: Gran Canaria	100	0.1
Shewanella algae	SY1	69	4,783,566	53	2023	Seawater	China: sanya city	100	0.1
Shewanella algae	SY102	81	5,107,245	53	2023	Seawater	China: sanya city	100	0.3
Shewanella algae	SY103	47	4,782,351	53	2023	Seawater	China: sanya city	100	0.2
Shewanella algae	SY104	40	4,782,630	53	2023	Seawater	China: sanya city	100	0.3
Shewanella algae	SY11	61	4,993,026	53	2023	Seawater	China: sanya city	100	1.4
Shewanella algae	SY15	45	4,890,572	53	2023	Seawater	China: sanya city	100	2.6
Shewanella algae	SY17	62	4,919,540	53	2023	Seawater	China: sanya city	100	0.1
Shewanella algae	SY4	52	4,785,102	53	2023	Seawater	China: sanya city	100	0.1
Shewanella algae	SY5	50	4,933,061	53	2023	Seawater	China: sanya city	100	0.3
Shewanella algae	SY6	25	4,795,749	53	2023	Seawater	China: sanya city	100	0.1
Shewanella algae	SY9	24	4,795,820	53	2023	Seawater	China: sanya city	100	0.1
Shewanella algae	SYC	49	4,770,716	53	2014	Homo sapiens	Taiwan	100	0.2
Shewanella algae	SYCDC17SW02	87	4,762,367	53	2017	Homo sapiens	China	100	0.1
Shewanella algae	SYCDC17SW03	89	4,757,243	53	2017	Homo sapiens	China	100	0.1
Shewanella algae	SYCDC17SW04	90	4,759,249	53	2017	Homo sapiens	China	100	0.1
Shewanella algae	SYCDC17SW07	69	4,870,207	53	2017	Homo sapiens	China	100	0.1
Shewanella algae	SYCDC18SW04	66	4,821,134	53	2018	Homo sapiens	China	100	0.3
Shewanella algae	SYCDC18SW07	81	4,638,041	53	2018	Homo sapiens	China	100	0.4
Shewanella algae	SYCDC18SW10	87	4,676,695	53	2018	Homo sapiens	China	100	0.1
Shewanella algae	SYCDC19SW01	50	4,719,010	53	2019	Homo sapiens	China	100	0.1
Shewanella algae	SYCDC19SW04	93	4,819,358	53	2019	Homo sapiens	China	100	0.1
Shewanella algae	SYCDC19SW05	68	4,876,395	53	2019	Homo sapiens	China	100	0.1
Shewanella algae	SYCDC19SW06	63	4,876,409	53	2019	Homo sapiens	China	100	0.1
Shewanella algae	SYT2	37	4,782,532	53	2014	Oysters (Crassostrea gigas)	Taiwan	100	0.2
Shewanella algae	SYT3	80	4,823,421	53	2014	Seawater	Taiwan	100	0.2
Shewanella algae	SYT4	37	4,839,649	53	2014	Mollusca (Meretrix lusoria)	Taiwan	100	0.2
Shewanella algae	TH101	61	4,867,371	53	2023	Seawater	China	100	0.2
Shewanella algae	TUM17378	1	4,872,739	53	2014	Homo sapiens	Japan: Tokyo	100	0.2
Shewanella algae	TUM17379	1	4,909,921	53	2014	Homo sapiens	Japan: Tokyo	100	0.1
Shewanella algae	TUM17382	1	5,040,456	53	2015	Homo sapiens	Japan: Tokyo	100	0.3
Shewanella algae	TUM17383	1	4,921,266	53	2015	Homo sapiens	Japan: Tokyo	100	0.2
Shewanella algae	TUM17384	1	4,871,625	53	2015	Homo sapiens	Japan: Tokyo	100	0.2
Shewanella algae	TUM17386	1	4,911,443	53	2015	Homo sapiens	Japan: Tokyo	100	0.2
Shewanella algae	TUM4442	1	4,798,767	53	mising	Homo sapiens	Japan: Tokyo	100	0.1
Shewanella algae	TYL	84	4,782,368	53	2014	Homo sapiens	Taiwan	100	0.2
Shewanella algae	VGH117	2	4,919,668	53	2015	Homo sapiens	Taiwan	100	0.1
Shewanella algae	XD101	20	4,809,236	53	2023	Seawater	China	100	2.0
Shewanella algae	YHL	27	4,850,439	53	2014	Homo sapiens	Taiwan	100	0.1
Shewanella algae	YK-SH	63	4,947,034	53	2020	Rearing water	China	100	2.8
Shewanella algae	YTH	44	4,785,906	53	2014	Homo sapiens	Taiwan	100	0.2
Shewanella algae	YTL	60	4,785,826	53	2014	Homo sapiens	Taiwan	100	0.3
Shewanella algae	YZ101	49	4,782,982	53	2023	Seawater	China	100	0.2
Shewanella algae	YZ102	49	4,780,811	53	2024	Seawater	China	100	0.2
Shewanella algae	YZ103	44	4,778,534	53	2023	Seawater	China	100	0.2

Table S3. NCBI-derived *S. algae* genomes carrying additional antimicrobial resistance genes beyond the intrinsic resistome (highlighted in red).

Strain	Accesion Number	Mobilome	Polymyxins	Multidrug resistance			β-lactams							
			ugd	MexI	CRP	rsmA	OXA-55/OXA-729/OXA-964	OXA-1	OXA-10	CTX-M-15	BRP(MBL)	VEB-1	CARB-3	NDM-1
6	JBDIMN010000006.1_1	Genomic Island	x	x	x	x	x							
150735	CP068229.1_671	Genomic Island	x	x	x	x	x							
HN_2022	JAODON010000013.1_76	Genomic Island	x	x	x	x	x							
502R	JBBXMQ010000004.1_2	Genomic Island	x	x	x	x	x							
13	JBDIMQ010000001.1_177	Genomic Island	x	x	x	x	x	x						
CHL	LVDF01000001.1_5	Genomic Island	x	x	x	x	x							
ATCC49138	AP024609.1_610	Integron	x	x	x	x	x							
S19	JBDINK010000011.1_55	Plasmid	x	x	x	x	x						x	
S7	JBDIMY010000002.1_336	Plasmid	x	x	x	x	x							
CCU101	CP018456.1_22	Plasmid	x	x	x	x	x							
VGH117	CP032415.1_1003	Plasmid	x	x	x	x	x							
CCU4051	CP078513.1_22	Plasmid	x	x	x	x	x							
CCU4052	CP078515.1_1080	Plasmid	x	x	x	x	x							
CCU4053	CP078518.1_1003	Plasmid	x	x	x	x	x							
CCU4054	CP078519.1_602	Plasmid	x	x	x	x	x							
KC-Na-R1	CP033575.1_940	Plasmid	x	x	x	x	x		x			x		
18064-CSB-B-B	CP047422.1_49	Plasmid	x	x	x	x	x			x				
S12	JBDIND010000014.1_52	Plasmid	x	x	x	x	x				x			x

Strain	Aminoglycosides										Tetracyclines	
	APH(3")-Ib	APH(6)-Id	aadA2	aadA3	aadA7	aadA16	AAC(6')-Ib8	AAC(3)-IId	armA	ANT(2")-Ia	tet(A)	tet(59)
6	x	x		x							x	
150735	x	x										x
HN_2022	x	x									x	
502R	x	x									x	
13	x	x	x				x				x	
CHL	x										x	
ATCC49138					x							
S19				x		x					x	
S7	x	x									x	
CCU101	x	x									x	
VGH117	x	x									x	
CCU4051	x	x									x	
CCU4052	x	x									x	
CCU4053	x	x									x	
CCU4054	x	x									x	
KC-Na-R1						x				x	x	
18064-CSB-B-B									x			
S12	x	x						x			x	

Strain	Sulfonamides / Trimethoprim						Phenicols		Quinolones		Macrolides / Lincosamides		Rifamycins		Others	
	sul1	sul2	dfrA1	dfrA12	dfrA15b	dfrA27	floR	cmlA5	QnrA3/QnrA4/QnrA7	QnrA1	mphA	Mrx	arr-2	arr-3	ugd	qacE
6	x	x					x		x						x	x
150735		x					x		x						x	
HN_2022		x					x		x						x	
502R		x			x		x		x						x	
13		x	x				x		x						x	
CHL		x					x		x						x	
ATCC49138	x								x						x	x
S19	x						x		x	x	x	x			x	
S7		x					x		x						x	
CCU101		x					x		x						x	
VGH117		x					x		x						x	
CCU4051		x					x		x						x	
CCU4052		x					x		x						x	
CCU4053		x					x		x						x	
CCU4054		x					x		x						x	
KC-Na-R1	x					x		x	x	x			x	x	x	x
18064-CSB-B-B	x			x					x						x	x
S12	x	x					x			x	x	x			x	x
